# Pan-cancer and single-cell analysis of actin cytoskeleton genes related to disulfidptosis

**DOI:** 10.1515/med-2024-0929

**Published:** 2024-03-30

**Authors:** Li-ping Shen, Han-tao Jiang

**Affiliations:** Department of Clinical Laboratory, Taizhou Hospital of Zhejiang Province affiliated to Wenzhou Medical University, Taizhou, 318000, Zhejiang Province, China; Department of Orthopedics, Taizhou Hospital of Zhejiang Province affiliated to Wenzhou Medical University, Taizhou, 318000, Zhejiang Province, China

**Keywords:** disulfidptosis, actin cytoskeleton, pan-cancer, single cell

## Abstract

Disulfidptosis was recently reported to be caused by abnormal disulfide accumulation in cells with high SLC7A11 levels subjected to glucose starvation, suggesting that targeting disulfidptosis was a potential strategy for cancer treatment. We analyzed the relationships between gene expression and mutations and prognoses of patients. In addition, the correlation between gene expression and immune cell infiltration was explored. The potential regulatory mechanisms of these genes were assessed by investigating their related signaling pathways involved in cancer, their expression patterns, and their cellular localization. Most cancer types showed a negative correlation between the gene-set variation analysis (GSVA) scores and infiltration of B cells and neutrophils, and a positive correlation between GSVA scores and infiltration of natural killer T and induced regulatory T cells. Single-cell analysis revealed that *ACTB*, *DSTN*, and *MYL6* were highly expressed in different bladder urothelial carcinoma subtypes, but *MYH10* showed a low expression. Immunofluorescence staining showed that actin cytoskeleton proteins were mainly localized in the actin filaments and plasma membrane. Notably, IQGAP1 was localized in the cell junctions. In conclusion, this study provided an overview of disulfidptosis-related actin cytoskeleton genes in pan-cancer. These genes were associated with the survival of patients and might be involved in cancer-related pathways.

## 1 Introduction

Despite advances in cancer treatment, the mortality of cancer is still high, and cancer remains one of the most serious health problems worldwide [1]. Therefore, there is a clear urgency to develop early and effective cancer management. Cancer databases offer valuable information on gene expression, genetic changes, immune infiltration, and clinical outcomes across various cancer types. Such data can aid in investigating the underlying mechanisms of cancer development and reveal potential targets for therapy [2].

Disulfidptosis, a previously undescribed form of cell death distinct from cuproptosis and ferroptosis, was recently reported to be caused by abnormal intracellular disulfide accumulation in cells with high SLC7A11 levels subjected to glucose starvation [3]. These experiments revealed the actin cytoskeleton’s sensitivity to disulfide stress-mediated disulfidptosis, suggesting that this process represents a potential therapeutic strategy for targeting disulfidptosis in patients with cancer. This article reveals a new cell death pathway associated with all cancers and investigates the potential role that actin cytoskeleton and disulfidptosis-related genes can play in cancer prognosis and treatment. The study focused on the 14 core genes involved in actin cytoskeleton genes related to disulfidptosis: *CD2AP*, *INF2*, *PDLIM1*, *ACTN4*, *MYH9*, *MYH10*, *IQGAP1*, *DSTN*, *ACTB*, *CAPZB*, *MYL6*, *TLN1*, *FLNA*, and *FLNB*. The actin cytoskeleton-related functions of these genes are presented in Table 1. Disulfidptosis is a metabolically related form of cell death that offers new directions for selectively targeting cancer metabolism to kill cancer cells [4]. Therefore, research on disulfidptosis has been rapid and extensive; nevertheless, disulfidptosis research is still in its infancy, and the specific mechanisms of this process are not yet fully understood. In addition, metabolic treatment of cancer cells by disulfidptosis may also affect immune cells. Therefore, the present study provides a timely, comprehensive summary of actin cytoskeleton-related genes in all cancer types along with the tumor immune microenvironment and lays the foundation for future studies to discover the role of disulfidptosis-related cell death in cancer. This discovery will provide clues for further clinical applications.

**Table 1:** Summary of actin cytoskeleton and disulfidptosis-related cell death

Gene symbol	Full name	Function in actin cytoskeleton	References
ACTB	Beta-actin	Regulation of actin cytoskeleton	[5]
ACTN4	Alpha-actinin-4	Regulation of actin cytoskeleton structure and function	[6]
CAPZB	F-actin capping protein subunit beta	Increasing actin filament depolymerization and capping	[7]
CD2AP	CD2-associated protein	Organizing the actin cytoskeleton at immunological synapses	[8]
DSTN	Destrin	Regulators involved in remodeling of the actin cytoskeleton	[9]
FLNA	Filamin A	Stabilization of the actin cytoskeleton	[10]
FLNB	Filamin B	Stabilization of the actin cytoskeleton	[10]
INF2	Inverted formin 2	Regulators involved in remodeling of the actin cytoskeleton	[11]
IQGAP1	IQ motif-containing GTPase activating protein 1	A fundamental regulator of cytoskeletal function	[12]
MYH9	myosin heavy chain 9	An important cytokine in cancer; involved in cytoskeletal reorganization	[13]
MYH10	myosin heavy chain 10	Associated with intracellular forces, organelle shuttling, cell adhesion, directional motility, and morphogenesis	[14]
MYL6	Myosin light chain 6	Bound by myosin heavy chain 14 and smooth muscle myosin	[15]
PDLIM1	PDZ and LIM domain protein 1	A cytoskeletal protein participating in cytoskeleton regulation and synapse formation	[16]
TLN1	Talin 1	Coordination of the actin cytoskeleton	[17]

## 2 Materials and methods

### 2.1 Gene-set enrichment analysis and gene-set variation analysis (GSVA)

All expression and plotting were based on the gene-set cancer analysis (GSCA, http://bioinfo.life.hust.edu.cn/GSCA/#/), an integrated platform for GSCA. The gene-set enrichment analysis provided a differential expression analysis of 14 cancer types (thyroid carcinoma [THCA], kidney renal papillary cell carcinoma [KIRP], bladder urothelial carcinoma [BLCA], liver hepatocellular carcinoma [LIHC], head and neck squamous cell carcinoma [HNSC], breast cancer [BRCA], lung adenocarcinoma [LUAD], prostate adenocarcinoma [PRAD], esophageal carcinoma [ESCA], kidney chromophobe [KICH], lung squamous cell carcinoma [LUSC], kidney renal cell carcinoma [KIRC], stomach adenocarcinoma [STAD], and colon adenocarcinoma [COAD]) with more than 10 paired tumor and normal samples. The fold change was calculated as the mean (tumor)/mean (normal); *p*-values were estimated by *t*-tests, *p* ≤ 0.05 was considered significant, and the false discovery rate (FDR) was further adjusted. The GSVA enrichment score measured how well genes were integrated into gene sets and positively correlated with their expression in each pathway. The GSCA estimated changes in the gene-set activity (denoted as the GSVA score) in a population of specific cancer samples in an unsupervised manner; these estimates were used for the GSVA analysis. The GSVA score was calculated with the R package GSVA.

### 2.2 Survival analysis

Using sample barcodes, median mRNA values, or GSVA scores, tumor samples were categorized into high-expression mRNA and low-expression mRNA, based on GSVA scores or mRNA expression. Based on the survival rate from the R package, both groups’ survival time and status were fitted. Additionally, we performed Cox proportional hazards analysis and conducted log-rank tests.

### 2.3 Identification of cancer subtypes

The expression and subtype analyses were used to find the expression of genes relevant to subtypes. This function utilized clinical data from nine cancer types (HNSC, LUSC, COAD, STAD, LUAD, GBM, BRCA, KIRC, and BLCA) in the GSCA. By sample barcoding, mRNA expression data were combined with clinical subtype information. The classification of cancer subtype required at least five samples. The Wilcoxon test was used to compare GSCA scores between two groups, when there were only two subtype groups. The analysis of variance (ANOVA) was used when there were more than two subtype groups.

### 2.4 Pathologic stages of cancers

The GSCA analyzed 9,478 tumor samples from 27 different types of cancer, including adenoid cystic carcinoma, BLCA, BRCA, cervical squamous cell carcinoma (CESC), cholangiocarcinoma, COAD, diffuse large B-cell lymphoma (DLBC), ESCA, HNSC, KICH, KIRC, KIRP, LIHC, LUAD, LUSC, mesothelioma (MESO), ovarian (OV), pancreatic adenocarcinoma (PAAD), rectum adenocarcinoma, skin cutaneous carcinoma (SKCM), STAD, testicular germ cell tumors (TGCTs), THCA, thymoma (THYM), uterine corpus endometrial carcinoma (UCEC), uterine carcinosarcoma, and uveal melanoma. The pathologic staging was determined for all samples, which was then utilized for the expression and stage analysis. Using sample barcodes, we merged the data of mRNA expression and clinical stage. Each stage subgroup was required to have at least five samples. According to the pathologic, clinical, and Masaoka (for THYM only) staging systems, the tumor samples were classified into four stages (I, II, III, and IV). Stage I included stage I, IA, IB, and IC; stage II included stage II, IIA, IIB, and IIC; stage III included stage III, IIIA, IIIB, and IIIC; and stage IV included stage IV, IVA, IVB, and IVC. The International Germ Cell Cancer Collaborative Group staging system (for TGCT only) classified samples into good (*n* = 32), intermediate (*n* = 9), and poor (*n* = 2).

### 2.5 Analysis of pathway activity

Reverse-phase protein array (RPPA) is a high-throughput proteomics technique. From RPPA data obtained from the Cancer Proteome Atlas, we assessed the pathway activity score of 10 recognized cancer pathways [18] in 7876 samples. And 32 cancer types from the Cancer Genome Atlas Program (TCGA) database were analyzed in the GSCA. To measure protein levels comparatively, RPPA data were normalized using the sample’s standard deviation and adjusted to align with the median value. The pathway score is then calculated as the total of the relative protein levels of all positive regulatory components in a given pathway minus those of negative regulatory components. According to the median gene expression, the samples were classified into two groups: high expression and low expression. The difference in the pathway activity score (PAS) between groups was detected using a Student’s *t*-test, for which the *p*-value was corrected with the FDR; the difference was considered statistically significant if FDR ≤0.05. If PAS (gene A high expression) > PAS (gene A low expression), gene A may have an activated effect on the pathway; otherwise, it may have a suppressive effect on the pathway.

### 2.6 Immune infiltration analysis

The study utilized ImmuCellAI to assess the infiltration of 24 immune cells. The correlation between immune cell infiltration and mRNA expression levels was determined through Spearman correlation analysis, with resulting *p*-values being adjusted through FDR. These analytical methods provide a comprehensive understanding of immune cell dynamics within the experimental setting, contributing to a more complete understanding of the study’s results.

### 2.7 Analysis of single-nucleotide variation (SNV) and copy number variation (CNV)

This section described the process of collecting SNV data from 10,234 cancer samples of 33 types in the TCGA database. In this analysis, deleterious and nondeleterious mutations were included. Deleterious mutations consist of missense, nonsense, frameshift insertions, splice sites, frameshift deletions, in-frame deletions, and in-frame insertions. Nondeleterious mutations consist of silent, intron, intergenic regions, 3′UTR, 5′UTR, 3′Flank, and 5′Flank. To perform the survival analysis, SNV and clinical data were integrated using sample barcodes, and samples with harmful mutations in specific genes were classified as the mutant group for further analysis. Groups with over two samples were included in the survival analysis. Gene-set SNV calculation was carried out to determine the status of the input gene set for each sample in the study. Samples could only be classified as the mutation group if the input gene set contained at least one mutant gene. This was necessary to accurately categorize samples into mutation groups. If all genes in the input gene set had no SNVs, the sample was categorized into the WT group. Only samples with deleterious mutants were included in the mutant group. The immune infiltration and SNV module estimated the difference in immune cell infiltration or the gene-set SNV levels between mutant and WT groups through the Wilcoxon test.

The CNV data were downloaded from the TCGA database and processed by GISTIC2.0 to identify regions with significant alterations in amplification or deletions in the patient group. Additionally, Spearman’s correlation was conducted to determine the correlation between CNV and gene mRNA expression. The samples were grouped into three categories – WT, Amp, and Dele, with the *p*-value of the survival analysis being obtained from groups with at least two samples. Furthermore, gene-set CNV was calculated to indicate the integrated CNV status of the input gene set for each sample. Samples were classified into the Amp or Dele groups if at least one gene in the input gene set was consistently amplified or deleted in that particular sample. In contrast, samples without any CNV in all genes within the input gene set were classified as part of the WT group. It is worth noting that in the case of inconsistent CNV status, whereby gene A was amplified while gene B was deleted, the sample would be classified as part of the excluded group, which was not included in this analysis. An immune infiltration and CNV module were utilized to estimate the relationship between the gene CNV and immune cell infiltration through a Spearman correlation analysis. To assess the potential correlation between immune cell infiltration and gene-set CNV, a statistical comparison of the mean infiltration levels among different gene-set CNV groups was conducted via the Wilcoxon test (for two groups) or one-way ANOVA (for more than two groups). These analyses provide insights into the potential interplay between genome-wide structural alterations and immune cell infiltration. Lastly, the *p*-value was adjusted by the FDR to ensure confidence in the results obtained from these analyses.

### 2.8 Methylation of disulfidptosis-related genes

Level 3 Illumina Human Methylation 450K data were downloaded from the TCGA database. A total of 14 cancer types (including THCA, KIRP, BLCA, LIHC, HNSC, BRCA, LUAD, PRAD, ESCA, KICH, LUSC, KIRC, STAD, and COAD), each with more than 10 pairs of tumor and adjacent non-tumor samples, were selected for analysis. Before conducting differential methylation analysis, correlation analysis was utilized to eliminate methylation sites that exhibited the strongest negative correlation with gene expression. A *t*-test was utilized to compute the *p*-value. The tumor samples were classified into hypermethylated or hypomethylated groups based on median methylation levels. Spearman correlation analysis was conducted to estimate the relationship between mRNA expression, immune cell infiltration, and methylation levels. To account for multiple tests, the *p*-value was adjusted using FDR.

### 2.9 Expression of disulfidptosis-related genes/proteins at the single-cell level

Single-cell analysis was performed (https://www.home-for-researchers.com/) to evaluate differences in disulfidptosis-related gene expression at the single-cell level. We employed Seurat, an R package, to execute quality control on pan-cancer data. Principal component analysis was utilized to decrease data dimensionality, followed by clustering cells with the FindClusters function. To identify cells, infer CNV and copycat, two R packages, were employed. We used UMAP to visualize data in reduced dimensions, while vlnplot, dimplot, and feature plot were used to demonstrate disulfidptosis-related gene levels in various cancers. Single-cell sequencing datasets of BLCA (GSE130001) and HNSC (GSE1163872) were obtained from the Gene Expression Omnibus database (https://www.ncbi.nlm.nih.gov/geo/) and the Single Cell Portal platform (http://singlecell.broadinstitute.org) for analysis. Overall, our approach provided a streamlined and effective means to process and analyze multi-dimensional single-cell data.

Immunohistochemistry images of disulfidptosis-related protein expression were downloaded from The Human Protein Atlas (HPA) to evaluate differences in disulfidptosis-related protein expression at the cellular level. The antibodies of the disulfidptosis-related proteins were ACTN4 (HPA006035), CAPZB (HPA031531), CD2AP (HPA003326, HPA003267), FLNA (HPA002925), FLNB (CAB019322), INF2 (HPA000724), IQGAP1 (CAB013302), MYH9 (HPA001644), MYH10 (HPA047541), TLN1 (HPA004748), PDLIM1 (CAB072840), and DSTN (HPA077782).

## 3 Results

### 3.1 Expression and related biological processes of disulfidptosis-related genes in cancers

**Figure 1:**
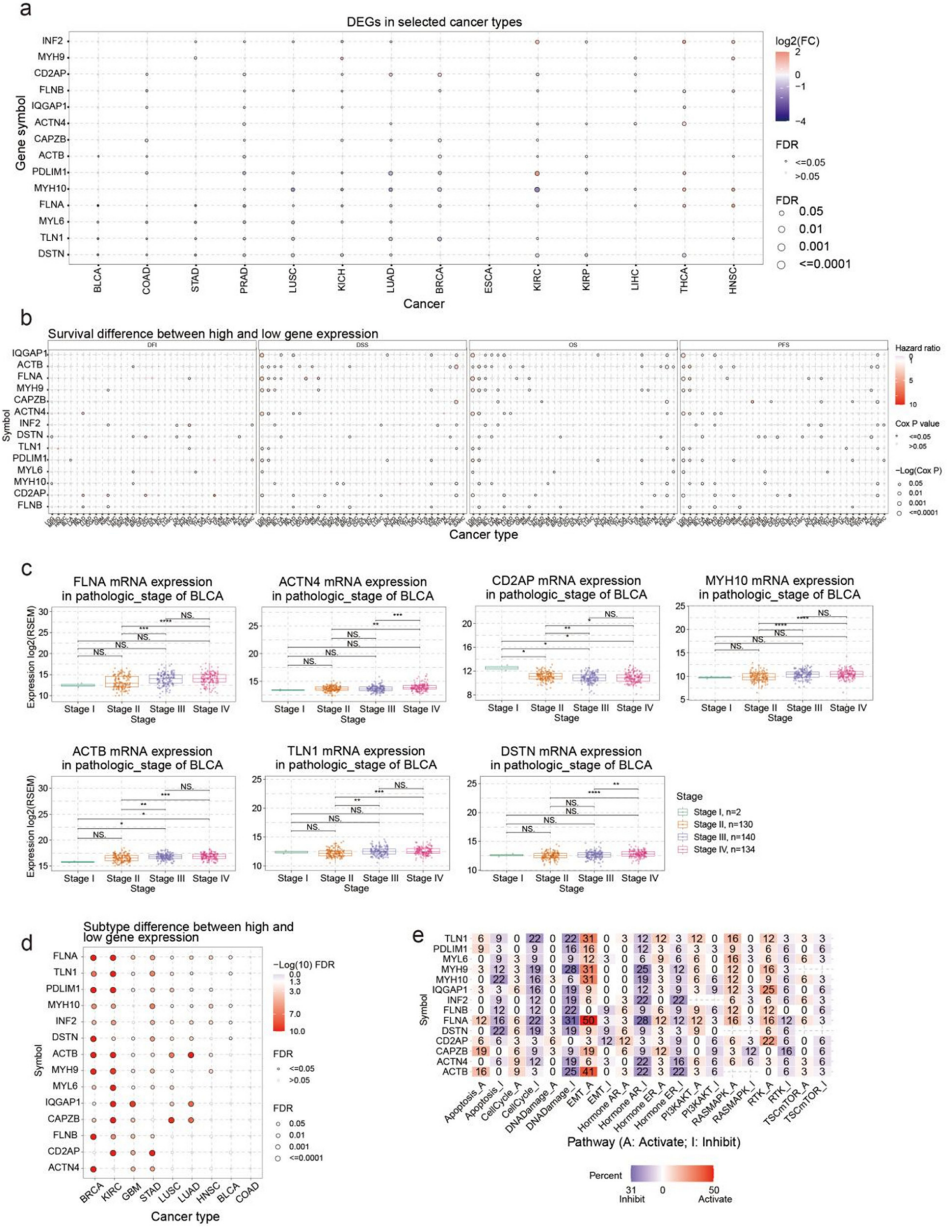
The correlation between disulfidptosis-related genes expression level with subtype, survival, pathological stage, and pathway activity. (a) The differentially expression genes between tumor and normal samples in the selected cancers; (b) the survival difference between high and low gene expression groups; (c) the difference of mRNA expression between stages in the specific cancers; (d) the associations between subtypes and gene expression; and (e) the percentage of cancers in which specific gene’s mRNA expression has a potential effect on pathway activity.

This study first outlined the expression profiles of disulfidptosis-related genes in cancer. A comparison of the expression of these genes in cancer and normal tissue revealed that disulfidptosis-related genes were expressed differently in different cancer types (i.e., they showed overexpression in some cancer types and low expression in others) (Figure 1a). For example, MYH10 was lowly expressed in KIRC, BRCA, LUAD, and LUSC but overexpressed in THCA and HNSC. Low expression of PDLIM1 was observed in LUAD, BRCA, and PRAD, but it was overexpressed in KIRC, THCA, and KIRP. THCA and HNSC tissues seemed to have higher expression levels of disulfidptosis-related genes compared with normal tissue. DSTN and FLNB were downregulated when *ACTIN4*, *INF2*, *MHY10*, *FLNA*, *PDLIM1*, and *IQGAP1* were upregulated in THCA cancer tissues. *MHY9*, *INF2*, *FLNA*, *MYH10*, *FLNB*, *FLN1*, and *ACTB* were upregulated in HNSC cancer tissues. Conversely, COAD and PRAD cancer tissues seemed to show lower levels of disulfidptosis. Except for *CD2AP*, *MYH9*, *MYH10*, and *FLNB*, disulfidptosis-related genes including *CAPZB*, *PDLIM1*, *MYL6*, *FLNB*, *IQGAP1*, *CD2AP*, *TLN1*, *DSTN*, and *ACTB* were downregulated in PRAD cancer tissues. We examined the link between disulfidptosis-related genes and clinical outcomes such as disease-free interval, disease-specific survival, overall survival, and progression-free survival. Our analysis revealed that disulfidptosis-related genes were associated with higher survival rates in LGG, MESO, and KIRC, as depicted in Figure 1b and Table S1. These findings have significant implications for understanding the role of these genes in the prognosis of these cancers. MESO patients with high *ACTH4*, *ACTB*, *PDLIM1*, *MHY9*, *MHY10*, *FLNB*, *FLNA*, and *DSTN* expressions had worse prognoses. LGG patients with high *CD2AP*, *IQGAP1*, *FLNA*, and *ACTN4* expressions had worse outcomes (Figure S1a). BRCA and THCA were the most significant cancer types in the analysis of pathological staging differences (Figure S1b). *FLNA*, *MYH10*, *ACTB*, *TLN1*, and *DSTN* expressions were higher in stages III and IV of BRCA than those in stages I and II. By contrast, *CD2AP* expression was lower in stages III and IV than in stages I and II (Figure 1c). The difference in disulfidptosis-related gene expression was most significant in different subtypes of BRCA and KIRC (Figure 1d, Table S2). Furthermore, we analyzed the potential impact of disulfidptosis-related proteins on established cancer pathways. Our findings revealed that all disulfidptosis-related genes exhibited potential involvement in signaling pathways associated with cancer, including the androgen receptor (AR), estrogen receptor (ER), PI3K/AKT, RAS/MAPK, receptor tyrosine kinase, cell cycle, and epithelial-to-mesenchymal transition (EMT) pathways. Most pathways were affected by *FLNA*, *ACTB*, and *TLN1*, and these proteins were potentially involved in the activation of the EMT and inhibition of DNA damage and the cell cycle. Notably, *FLNA* was associated with EMT activation in 50% of cancers (Figure 1e).

### 3.2 Immune profile of disulfidptosis-related genes in cancers

**Figure 2:**
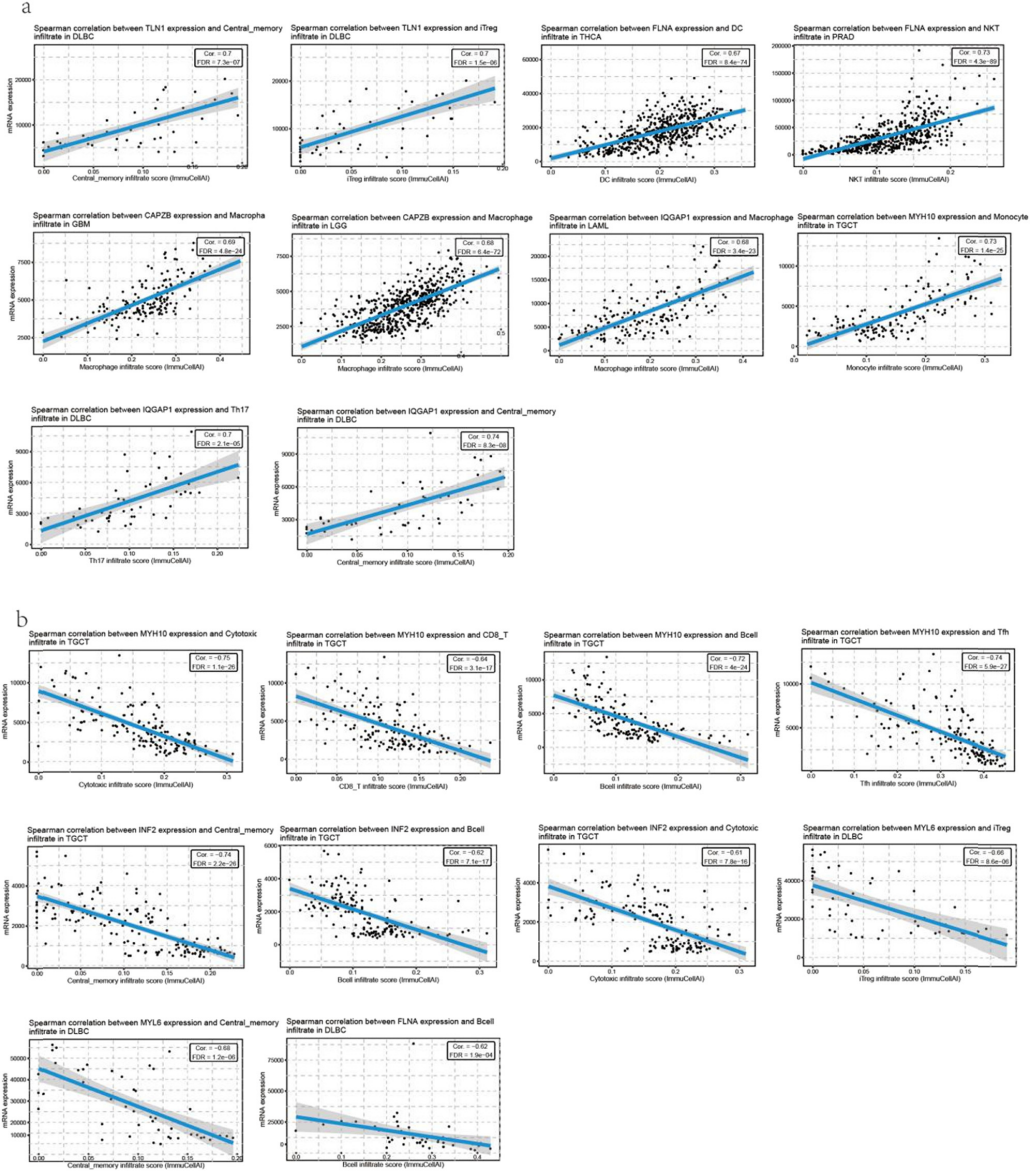
The correlation between immune infiltration with disulfidptosis-related genes expression. (a) The top 10 positive correlation among inputted gene set in specific cancers and (b) the top 10 negative correlation among inputted gene set in specific cancer.

Most immune cells showed significant correlations with the level of disulfidptosis-related gene expression. The 10 most positive correlations were as follows: increased *TLN1* expression was associated with increased central memory cell infiltration and induced regulatory T (iTreg) infiltration in DLBC; increased *FLNA* expression was associated with increased dendritic cell (DC) infiltration in THCA; increased *FLNA* expression was associated with increased natural killer T (NKT) infiltration in PRAD; increased *CAPZB* expression was associated with increased macrophage infiltration in GBM and LGG; increased *IQGAP1* expression was associated with increased macrophage infiltration in LAML; increased *TH17* expression was associated with increased central memory infiltration in DLBC; and increased *MYH10* expression was associated with increased monocyte infiltration in TGCT (Figure 2a, Table S3). The top 10 negative correlations were as follows: decreased *MYH10* expression was associated with decreased cytotoxic infiltrate, CD8^+^ T infiltration, B-cell infiltration, and Tfh infiltration in TGCT; decreased *INF2* expression was associated with decreased central memory infiltration, B-cell infiltration, cytotoxic infiltration, and iTreg infiltration in TGCT; and decreased *MYL6* expression was associated with central memory infiltration and B-cell infiltration in DLBC (Figure 2b, Table S3).

### 3.3 GSVA score of disulfidptosis-related gene set in cancers

**Figure 3:**
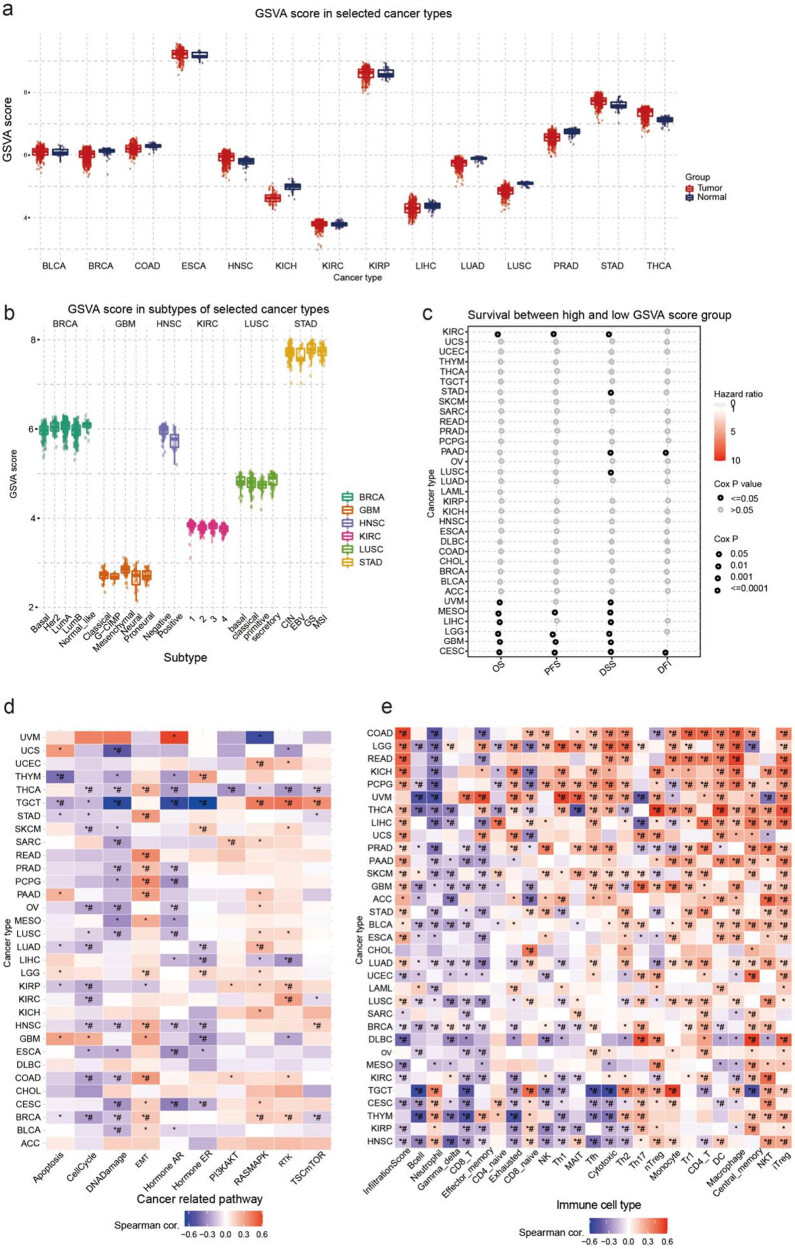
The correlation between gene set expression (GSVA) score with immune infiltration, cancer subtypes, and survival. (a) Box plot compares the GSVA score between tumor and normal samples and (b) Box plot presents the GSVA score among subtypes in the selected cancers. (c) The results of survival difference between GSVA score groups in selected cancers; (d) the association between GSVA score and activity of cancer-related pathways in selected cancers; and (e) the association between GSVA score and immune cell infiltration in selected cancers.

The three most significant differences in GSVA scores between tumor and normal tissues were found in LUSC, THCA, and LUAD. The GSVA score of THCA tumors was higher than that of normal tissue, whereas the GSVA scores of LUSC and LUAD were lower than those of normal tissue (Figure 3a). In the analysis of differences in GSVA scores between cancer subtypes, BRCA and KIRC showed the most significant differences; Basal and LumB showed lower GSVA scores than the normal-like subtype in BRCA, and the GSVA score of type 1 was the highest and that of type 4 was lowest in KIRC (Figure 3b). This section examined the correlation between GSVA scores and survival rates for 10 different types of cancer. Of these types, CESC, GBM, and MESO showed the most significant correlations (Figure 3c). We also analyzed the potential association between GSVA scores of disulfidptosis-related genes and cancer-related pathways. The pathway association network suggested that all disulfidptosis-related genes may play a role in cancer-related pathways, including cell cycle, DNA damage, EMT, AR, and ER pathways. Across most cancer types, a positive correlation was identified between GSVA scores and EMT and RAS/MAPK pathways, while a negative correlation was identified with cell cycle, DNA damage, and AR pathways (Figure 3d). Furthermore, we explored the relationship between disulfidptosis-related genes and the tumor immune microenvironment. To analyze this, we created a gene-set signature (GSVA score) synthesizing disulfidptosis-related genes to analyze their correlation with immune cell infiltration. Results showed that GSVA scores were significantly correlated with the infiltration of multiple immune cells in various cancer types. In particular, GSVA scores demonstrated a negative correlation with B-cell and neutrophil infiltration while being positively correlated with NKT and iTreg cell infiltration in most cancer types (Figure 3e).

### 3.4 Expression of disulfidptosis-related genes at single-cell level

**Figure 4:**
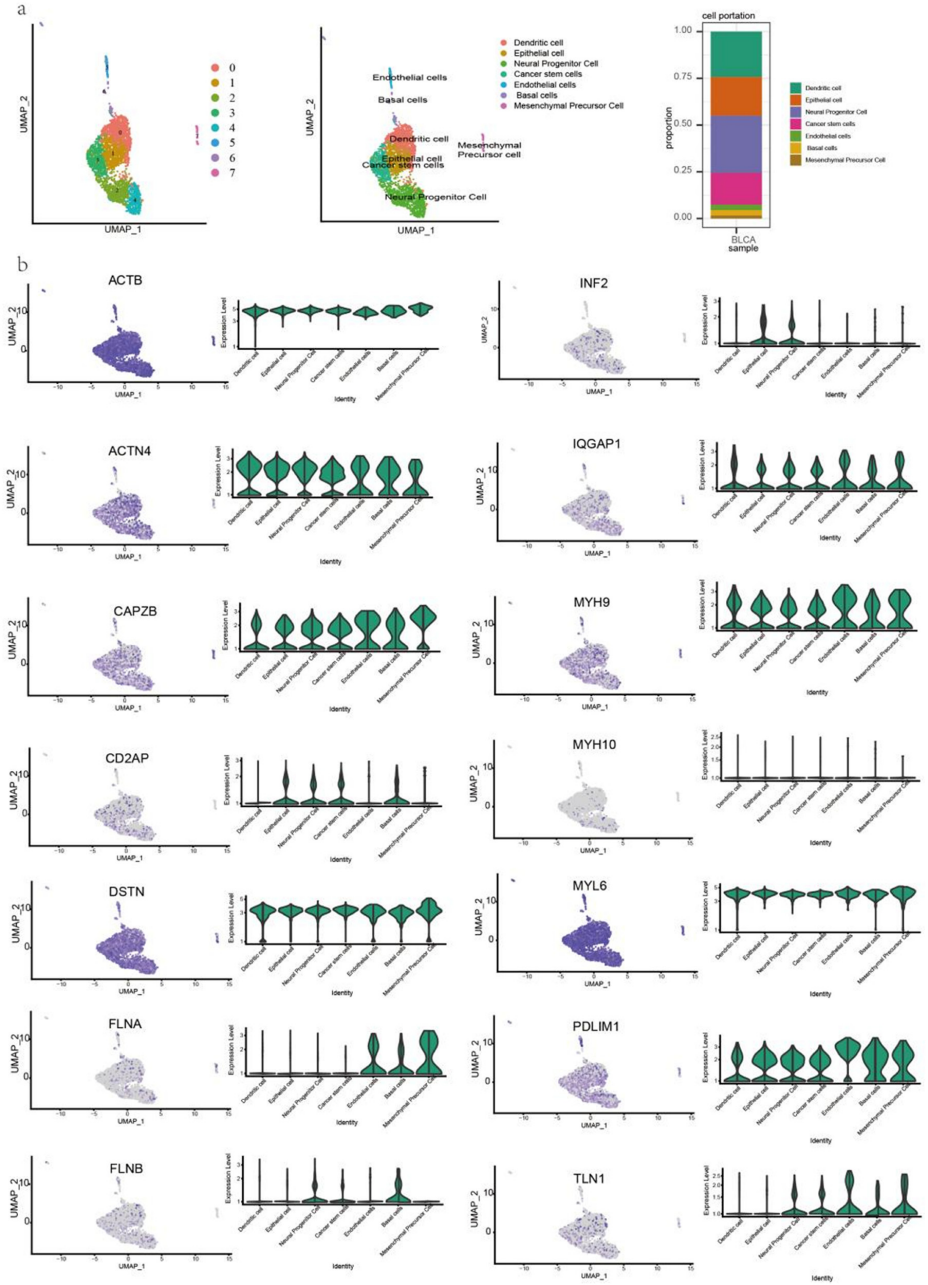
Single-cell sequencing analyzing the disulfidptosis-related genes expression in cells in BLCA. (a) Cells were grouped and identified and (b) the expression level of disulfidptosis-related genes in different cell clusters.

To analyze disulfidptosis-related gene expression in different cell types, we performed a single-cell analysis in BLCA and HNSC. The cells were identified including dendritic, epithelial, neural progenitor, cancer stem, endothelial, basal, and mesenchymal precursor cells (Figure 4a). ACTB, DSTN, and MYL6 were highly expressed, whereas MYH10 was lowly expressed in the cell subsets of BLCA. Notably, FLNA was highly expressed in endothelial, basal, and mesenchymal precursor cells but lowly expressed in the other cell types. FLNB was highly expressed in neural progenitor and basal cells (Figure 4b). Moreover, monocyte, mesenchymal, neural progenitor, and muscle cells were identified in HNSC (Figure S2a). ACTB, DSTN, and MYL6 were highly expressed, whereas CD2AP and INF2 were lowly expressed in all cell subsets. MYH10 was expressed at higher levels in the neural progenitor cells of HNSC, which had a different expression pattern than that in BLCA. Notably, CAPAB, FLNA, and FLN1 were more highly expressed in all cell subsets of HNSC than those of BLCA (Figure S2b).

Furthermore, we analyzed the metabolic pathways of different cell subsets. In BLCA and HNSC, the single-sample gene-set enrichment analysis revealed that neural progenitor cells had the highest score in pathways associated with tumor proliferation, such as the G2M checkpoint, DNA replication, and DNA repair pathways. By contrast, neural progenitor cells were lowly enriched with the EMT maker, extracellular matrix (ECM)-related genes, degradation of ECM, collagen formation, apoptosis, P53, inflammation signature, and angiogenesis pathways (Figure S3a and b).

**Figure 5:**
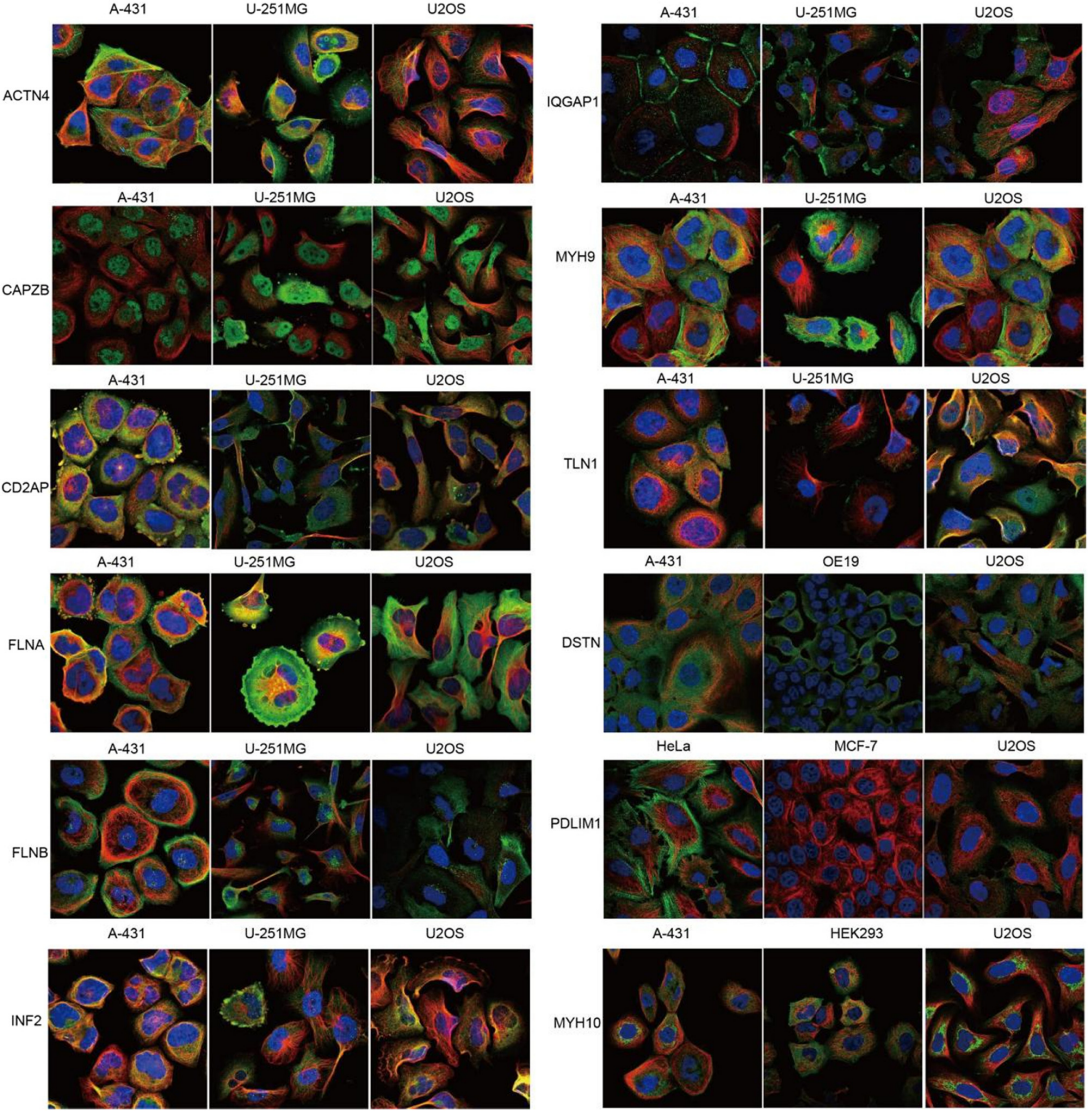
The expression location disulfidptosis-related proteins in human sub cells.

Then we analyzed the expression and localization of disulfidptosis-related protein expression at the subcellular level in the HPA database. The ACTN4, FLNA, MYH9, MYH10, and PDLIM1 proteins were mainly located in the actin filaments, whereas the CD2AP, FLNA, FLNB, IQGAP1, MYH9, and DSTN proteins were mainly located in the plasma membrane. The CAPZB and TLN1 proteins were mainly in the cytosol, and INF2 proteins were in the endoplasmic reticulum. Notably, IQGAP1 was located in the cell junctions (Figure 5).

### 3.5 SNV of disulfidptosis-related genes in cancers

**Figure 6:**
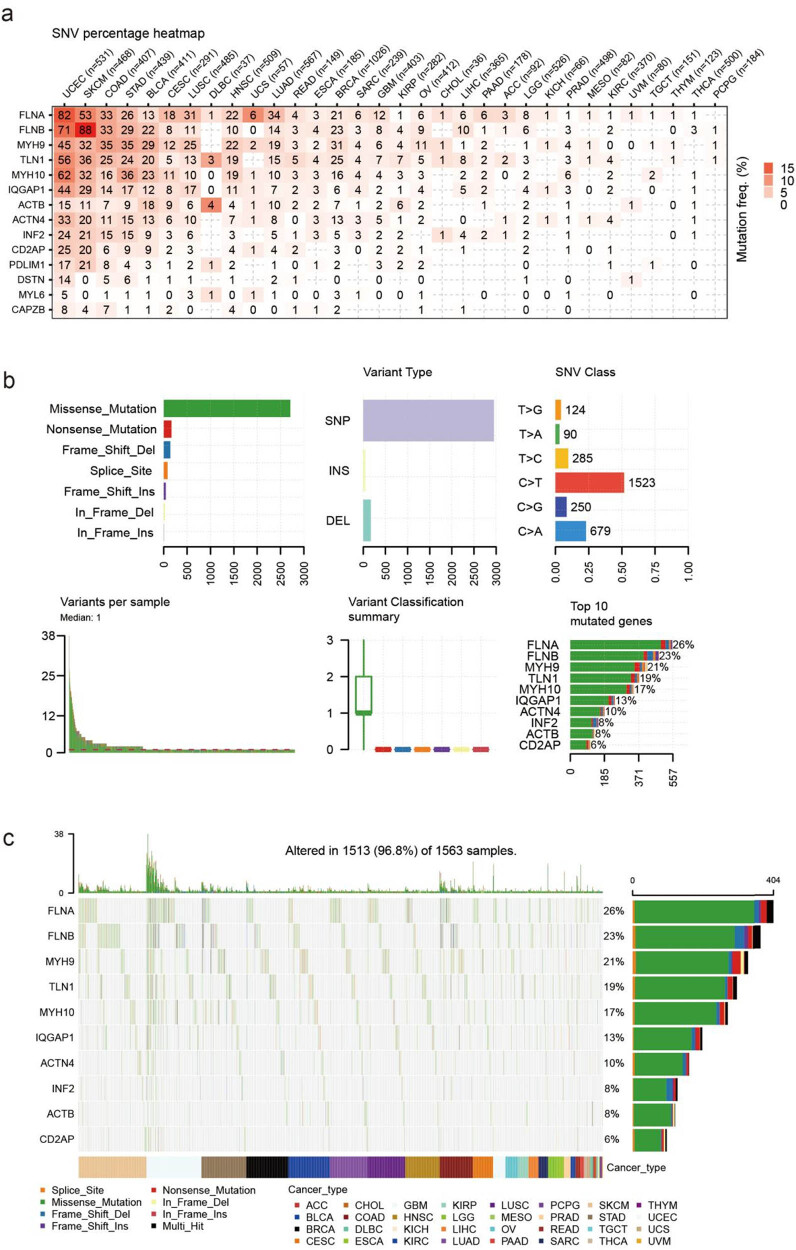
The SNV summary of disulfidptosis-related genes in pan-cancer. (a) The profile of SNV of the inputted gene set in the selected cancers; (b) the SNV classes of inputted gene set in the selected cancers; and (c) oncoplot provides the situation of the SNV of the top 10 mutated genes among inputted gene set in the specific cancers.

FLNA and FLNB were the most commonly mutated genes related to disulfidptosis in cancer, as revealed by the SNV analysis. For example, among 531 UCEC samples, 82 showed FLNA SNV, and among 468 SKCM samples, 53 showed FLNB SNV. These two cancer types also showed the most mutation in other disulfidptosis-related genes (Figure 6a). The statistical results showed that most of the SNVs were missense mutations, and single-nucleotide polymorphism was the most common form of genetic variation; the most common class of SNVs was C > T (Figure 6b). The SNV landscape map revealed that the SNV of FLNA, FLNB, and MYH9 was frequently detected in various cancers (Figure 6c).

**Figure 7:**
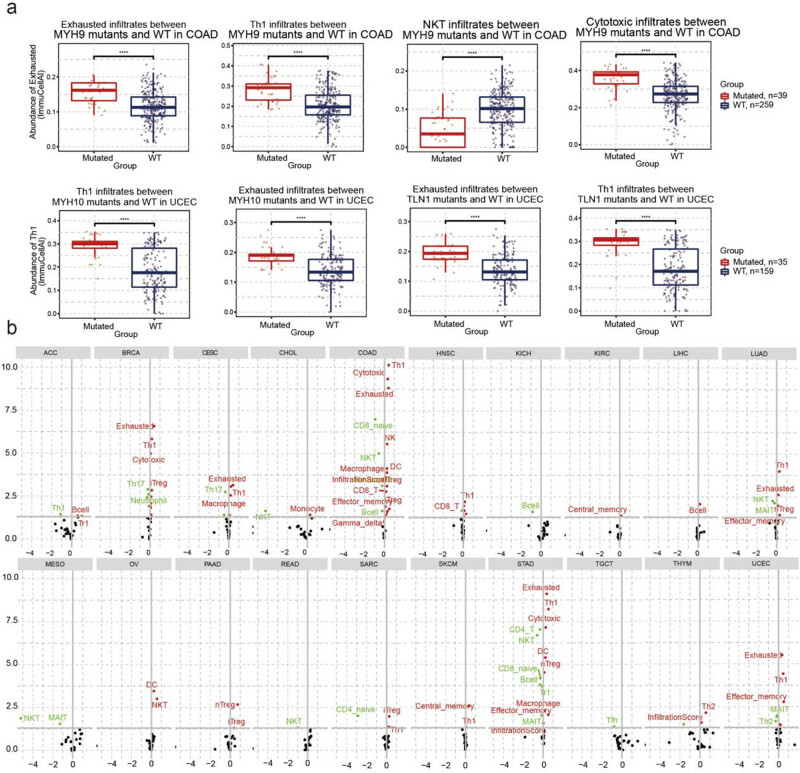
The correlation between disulfidptosis-related genes SNV with immune infiltration. (a) The difference of immune infiltration between mutant and wide type in COAD and uterine corpus endometrial carcinoma (USEC) and (b) the difference of immune infiltration between gene set SNV groups.

Additionally, we analyzed the relationship between immune infiltration and SNV. COAD patients with MYH9 mutations had higher exhausted T-cell, Th1, and cytotoxic T-cell infiltration but lower NKT infiltration than those in the WT group. UCEC patients with MYH10 mutations and TLN1 have higher exhausted T-cell and Th1 infiltration (Figure 7a, Table S4). In addition, the SNV of disulfidptosis-related genes was closely correlated with immune infiltration. Specifically, the SNV of disulfidptosis-related genes was positively correlated with Treg, DC, macrophage, effector memory, and exhausted T-cell infiltration but negatively correlated with Th17, NKT, CD4 naïve, CD8 naïve, and muscle-associated invariant T-cell infiltration. In COAD and STAD, the SNV of disulfidptosis-related genes had the highest association with immune cell infiltration (Figure 7b, Table S5).

In addition, we analyzed the relationship between SNVs of the disulfidptosis-related genes and the survival of patients. In UCEC, the SNVs of FLNB, TLN1, and FLNA were found to have a significant correlation with the overall survival. However, in most other cancers, SNVs of genes related to disulfidptosis were not significantly associated with patient survival (Figure S4a and b, Table S6).

### 3.6 CNV of disulfidptosis-related genes in cancers

**Figure 8:**
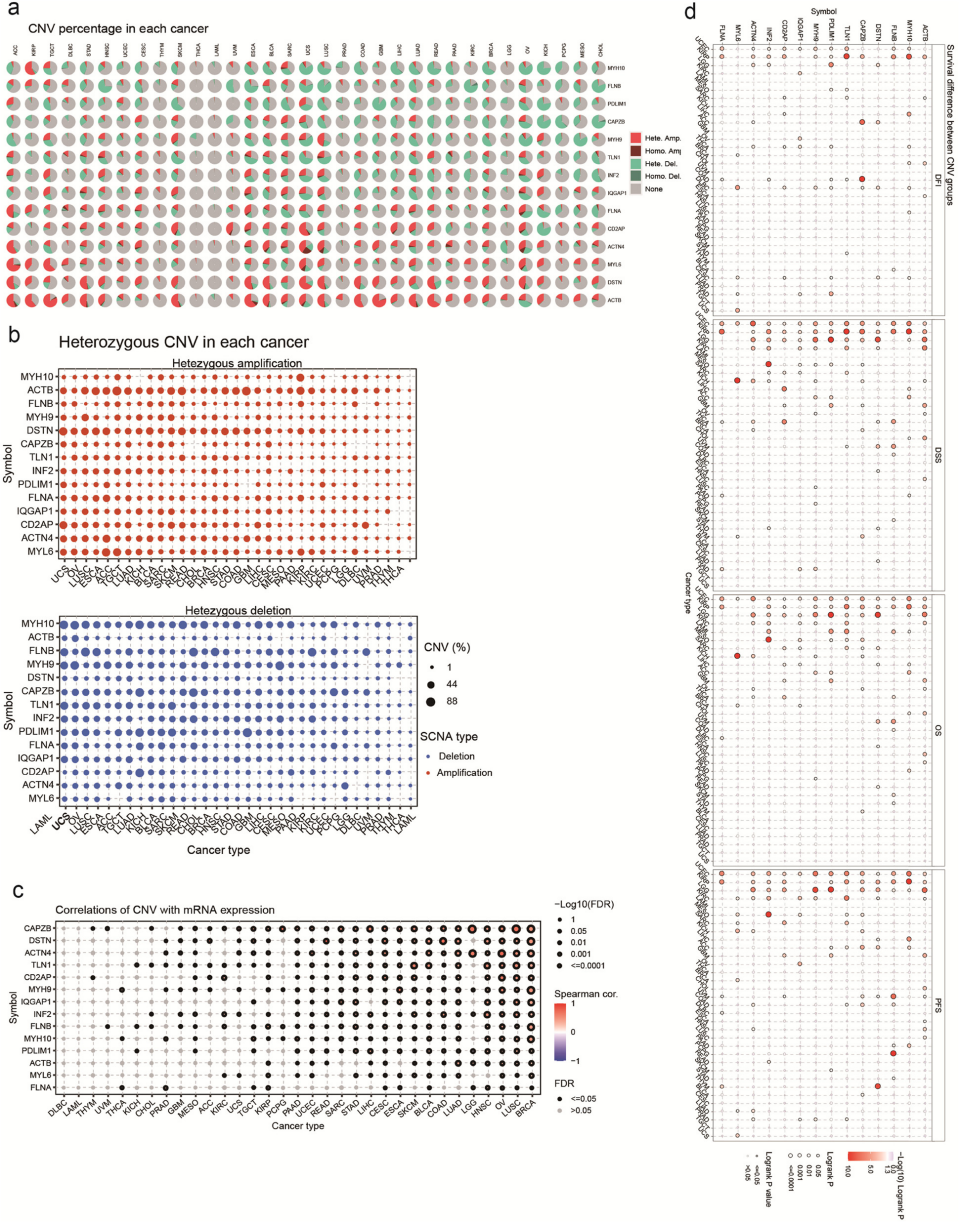
The CNV summary and its correlation with gene expression and survival. (a) The pie plot summarizes the CNV of inputted genes in the selected cancer types; (b) the profile of heterozygous CNV of inputted genes in the selected cancers; (c) the correlations between CNV and mRNA expression in the selected cancers; and (d) the difference of survival between CNV and wide type in the selected cancers.

To understand the potential factors influencing the expression of disulfidptosis-related genes in cancer, we conducted a comprehensive study that examined the CNV of these genes across different types of cancer. Our results revealed that CNVs exhibit distinct patterns across different cancer types, with heterozygous amplifications and deletions accounting for the most common types of CNVs (Figure 8a). Subsequently, we further analyzed the heterozygous amplifications and deletions of disulfidptosis-related genes, which provided crucial insights into the role of these genes in cancer pathogenesis (Figure 8b). Additionally, we assessed the impact of CNV on gene expression. We established that CNVs had significant correlations with corresponding gene expression, especially in BRCA, LUSC, OV, HNSC, and LGG (Figure 8c). Notably, our analysis of the correlation between CNV and survival in multiple cancer types revealed that CNV levels were associated with the survival of KIRP, UCEC, and LGG patients (Figure 8d). Furthermore, we analyzed the relationship between CNV of disulfidptosis-related genes and the survival of cancer patients. In UVM, the CNV of disulfidptosis-related genes was strongly correlated with patient survival (Figure S5a).

**Figure 9:**
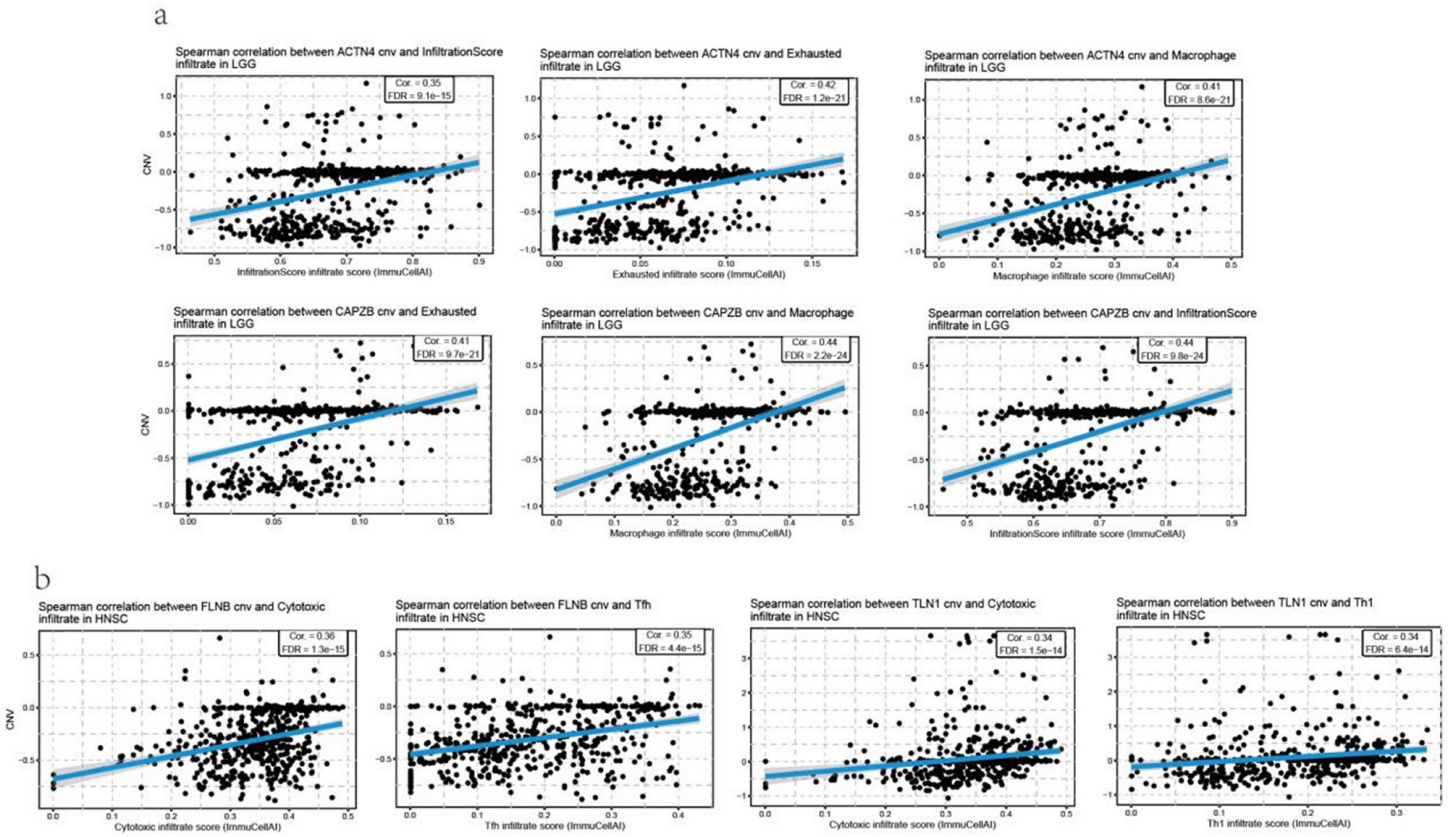
The correlation between disulfidptosis-related genes CNV with immune infiltration. (a) The correlation between immune infiltration with disulfidptosis-related genes CNV in brain LGG and (b) the correlation between immune infiltration with disulfidptosis-related genes CNV in head and neck squamous cell carcinoma (HNSC).

Additionally, a relationship between CNV and immune infiltration was analyzed, which was significantly correlated in most cancer types. *ACTN4* and *CAPZB* CNVs were positively correlated with the infiltration of exhausted T cells and macrophage in LGG (FIgure 9a). There was a positive correlation between *FLNB* CNV and cytotoxic T cells and Tfh infiltration in HNSC. In addition, there was a positive correlation between *TLN1* CNV and cytotoxic T-cell and Th1 infiltration in HNSC (Figure 9b, Table S7). Furthermore, the CNV of disulfidptosis-related genes was negatively correlated with Th17, NKT, CD4 naïve, and exhausted T-cell infiltration, especially in COAD and STAD (Figure S5b, Table S8).

### 3.7 Methylation of disulfidptosis-related genes in cancer

**Figure 10:**
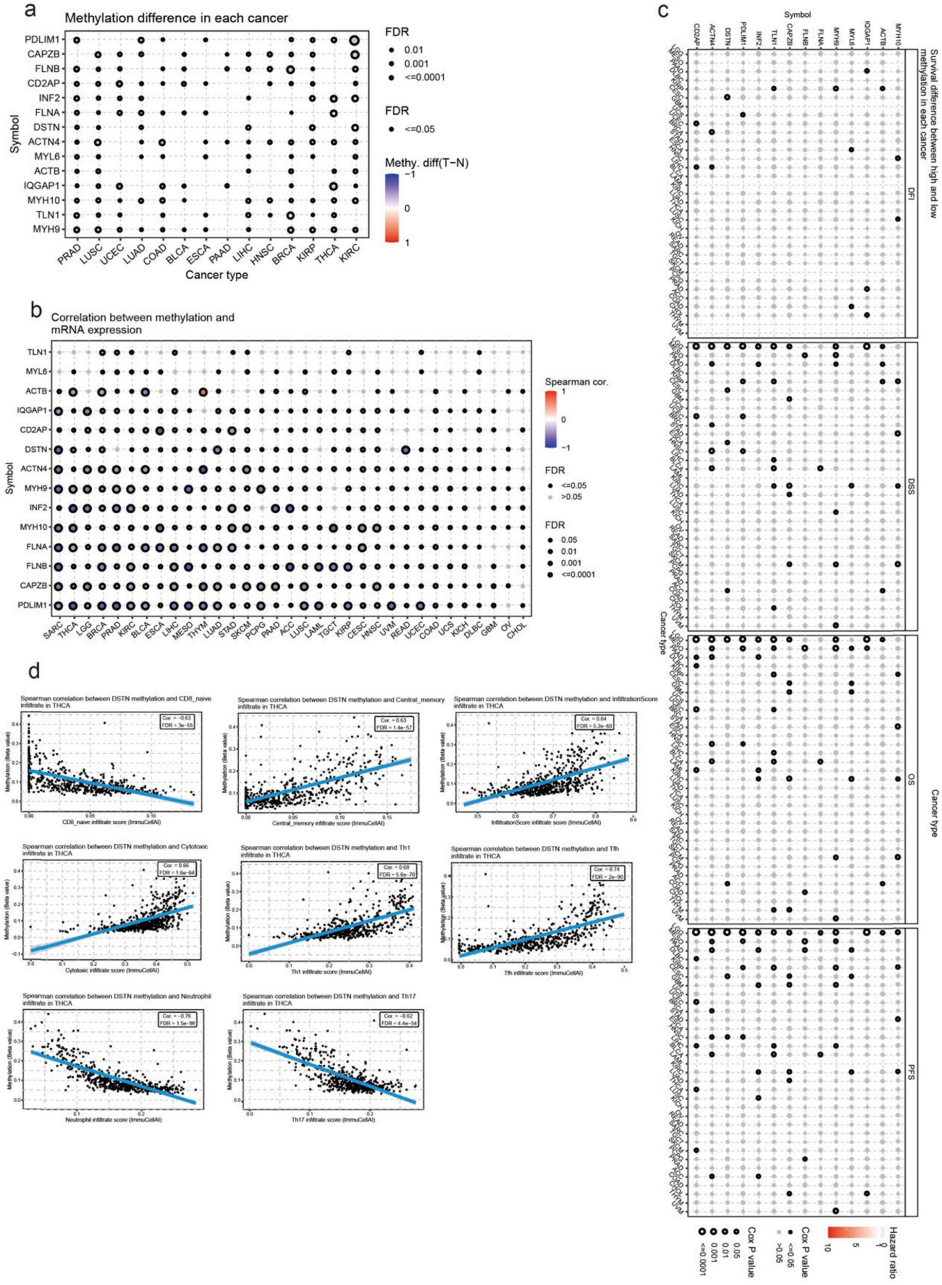
The methylation and its correlation with gene expression and survival. (a) The methylation difference between tumor and normal samples of inputted genes in the selected cancers; (b) the profile of correlations between methylation and mRNA expression of inputted genes in the specific cancers; (c) the overall survival difference between higher and lower methylation groups in the specific cancers; and (d) the correlation between gene methylation and immune infiltration in the specific cancers.

Another factor that may affect the expression of genes related to disulfidptosis is methylation. Therefore, we compared the differences in methylation levels between cancer and normal tissues. The cancer types with the most significant differences between cancer and normal tissues were KIRC, BRCA, and THCA (Figure 10a). Most cancer types showed a negative correlation between disulfidptosis-related genes and methylation, expected for *TLN1* and *MYL6*. Notably, the expression of *ACTB* was positively correlated with *THY* methylation (Figure 10b). When patients were classified into high- and low-methylation groups, we found that the methylation level of disulfidptosis-related genes was significantly associated with the survival of LGG and MESO patients (Figure 10c).

Furthermore, in most types of cancers, there was an association between immune infiltration and disulfidptosis-related genes. For example, a negative correlation was found between DSTN methylation levels and CD8 naïve, neutrophil, and Th17 infiltration in THCA. By contrast, a positive correlation was found between DSTN methylation levels and central memory, infiltration score, cytotoxic T-cell, Th1, and Tfh infiltration (Figure 10d, Table S9).

### 3.8 Association of disulfidptosis-related genes with drug sensitivity in cancer

**Figure 11:**
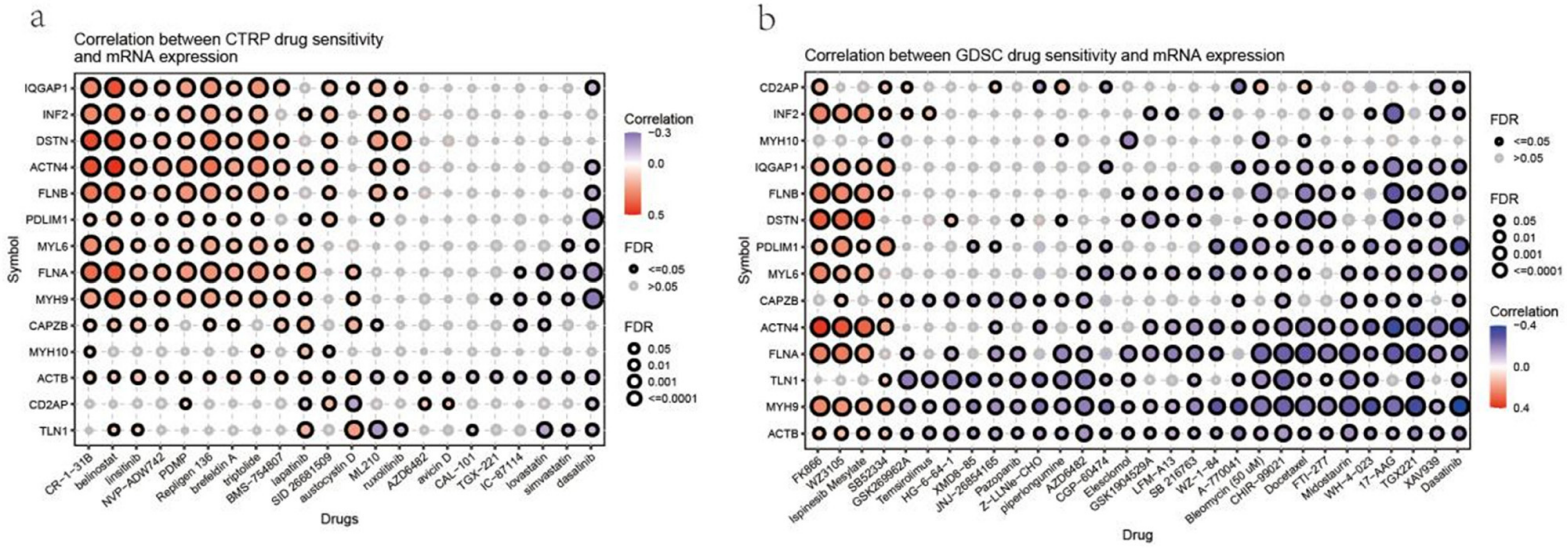
Information about the correlation between gene expression and CTRP, GDSC drug sensitivity in pan-cancer. (a) The correlation between gene expression and the sensitivity of CTRP drugs (top 30) in pan-cancer and (b) the correlation between gene expression and the sensitivity of GDSC drugs (top 30) in pan-cancer.

Furthermore, we used the Genomics of Drug Sensitivity in Cancer (GDSC) and Cancer Therapeutics Response Portal (CTRP) databases to characterize the association between disulfidptosis-related gene expressions and drug sensitivity in cancer cell lines. These databases provide the IC50 of various compounds on different cancer cell lines and describe the expression of genes in these cell lines. Using these data, we correlated gene expression with compound sensitivity in cell lines. The results indicated that the disulfidptosis-related genes were significantly correlated with the sensitivity of cancer cells to multiple compound. In the CTRP database, the expression of disulfidptosis-related genes was most positively associated with the sensitivity of drugs such as CR-1-31B, belinostat, and repligen 136 (top three) and negatively associated with the sensitivity of drugs such as dasatinib, simvastatin, and lovastatin (top three) (Figure 11a). In the GDSC database, the expression of disulfidptosis-related genes was most positively associated with the sensitivity of drugs such as FFK866, WZ3105, and ispinesib mesylate (top three) and negatively associated with the sensitivity of drugs such as dasatinib, XAV939, and TGX221 (top three) (Figure 11b).

## 4 Discussion

Disulfidptosis is a recently identified and described form of cell death. When SLC7A11-high cells are subjected to glucose starvation, they undergo NADPH depletion and an aberrant accumulation of intracellular cystine and other disulfide molecules (e.g., actin cytoskeleton proteins). This leads to disulfide stress, alterations in protein function, the collapse of the actin network, and detachment from the plasma membrane, resulting in disulfidptotic cell death. However, disulfidptosis does not exhibit common features of other known forms of regulated cell death, such as caspase-3 cleavage or ATP depletion [19,20]. NADPH depletion has been linked to several ROS-induced cell death processes, including apoptosis [21], necrosis [22], and pyroptosis [23]. Unusually, disulfidptosis in SLC7A11-high cells is characterized by NADPH depletion and enhanced cystine uptake. The use of pharmacologic and genetic approaches to block other cell death mechanisms does not stop this form of cell death. This suggests that SLC7A11-high tumors are likely resistant to therapies that induce ferroptosis and apoptosis and that this vulnerability of cancer cells to disulfidptosis offers new therapeutic opportunities for treating SLC7A11-high cancers. Studies suggested that disulfide stress represented a targetable metabolic pathway in cancer; disulfide-based prodrugs could be used for cancer treatment [3,24,25,26].

No previous studies have investigated the downstream genes of the disulfidptosis pathway; however, the efficacy of therapies targeting disulfidptosis may depend on downstream proteins. Therefore, further studies on these downstream proteins would be valuable for developing disulfidptosis-based therapies. Proteomic analyses in glucose-starved SLC7A11 high cells revealed that differentially expressed proteins were enriched in actin cytoskeleton processes, and there were 14 actin cytoskeleton proteins [3]. This study provides a comprehensive and systematic characterization of the actin cytoskeleton-related genes in different cancer types by assessing publicly available data and performing single-cell analysis. Our results revealed multiple mechanisms of disulfidptosis-related gene expression regulation in cancers and the potential links between disulfidptosis-related genes and other cancer-related pathways, thus providing a holistic picture of disulfidptosis-related genes in all types of cancer. By elucidating their pivotal involvement in cancer pathogenesis, progression, tumor microenvironment interactions, and drug resistance mechanisms, this research underscores the significant impact of these genes in oncology. Furthermore, the identification of these genes as potential prognostic and therapeutic biomarkers for diverse cancers not only highlights their clinical relevance but also opens new avenues for innovative research and treatment approaches in the field of cancer therapy.

The actin cytoskeleton and actin-binding proteins are involved in all stages of carcinogenesis. For example, actin-binding proteins play a role in the initial steps of carcinogenesis by regulating the expression of oncogenes; in addition, alterations in actin localization inhibit apoptosis and promote cell proliferation, and the formation of actin-rich protrusions regulate the migration and invasion of cancer cells [11,27,28,29]. Actin cytoskeleton proteins play an important role in tumor metastasis, including EMT activation, matrix adhesion, pseudopodia formation, and local invasion [5,30]. Similarly, the actin cytoskeleton plays a crucial role in promoting tumor angiogenesis [31]. For example, the most studied tumor-related actin cytoskeleton protein is IQGAP1, a scaffold protein that interacts with cytoskeletal components. It is highly expressed in gliomas [32,33], CRC [34], PRAD [35], THCA [36], BRCA [27], PAAD [33,37,38], ESCA [39], LIHC [33], HNSC [40], and other tumor tissues; it promotes the EMT, tumor cell proliferation and migration, and invasion through Wnt/β-linked protein signaling, NF-κB, endothelin-1 receptor signaling, MAPK, and other pathways, and it inhibits ECM degradation [41] and apoptosis [27]. However, our results showed no differences between IQGAP1 levels in LIHC or ESCA tissues and normal tissue. Previous studies have shown that IQGAP1 is associated with shorter overall survival in patients with BLCA [42], BRCA [27], CRC [34], UCEC [43], LUAD [44], GBMLGG [32], and HNSC [45]. Similarly, our results showed that high IQGAP1 expression was associated with unfavorable overall survival in patients with HNSC and LGG. Although some studies suggested that IQGAP1 was not frequently mutated in cancer apart from HNSC [46,47], the results of this study indicated that IQGAP1 exhibited high mutation rates in USEC, SKCM, COAD, STAD, BLCA, CESC, and LUSC.

The tumor immune microenvironment is inextricably linked to tumor development and tumor-targeted therapy. Natural killer (NK) cells are a major subset of intrinsic lymphocytes that exert immune surveillance against tumors by killing target cells and secreting a variety of cytokines. Actin is the structural basis for NK cell immune synapse (NKIS) and secretory apparatus polarization [48,49], and the rapid formation of plate-like pseudopods and pseudopods during immune cell migration requires large amounts of actin polymerization [50]. Therefore, the cytoskeleton can promote the effector functions of NK cells, such as motility, infiltration, binding to target cells, immune synapse assembly, and cytotoxicity. In this study, increased expression of PDLIM1, INF2, ACTB, CAPZB, MYL6, FLNA, TLN1, and CA2AP was significantly correlated with increased NK cell infiltration in specific cancers; notably, CD2AP levels showed a significant negative correlation with NK cell infiltration in PRAD. The functional shift of DCs from migration to antigen uptake and processing is also tightly regulated by actin dynamics, and the deletion of actin regulatory proteins affects DC migration as well as antigen processing and presentation [13]. The results of this study showed that FLNA, IQGAP1, MYH10, TLN1, PDLIM1, INF2, CAPZB, ACTN4, DSTN, and MYH9 were significantly positively associated with DC cell infiltration in specific cancers, with a significant negative correlation between DC cells in DSTN and THCA. In adaptive immune cells (specifically T cells), phosphorylated YAP regulates T-cell activation by modulating the binding affinity between NFAT1 (a transcription factor that regulates T-cell activation and metabolism) and its support protein, IQGAP1 (based on matrix stiffness) [51]. The migratory behavior of CD8 + T cells is regulated by their actin cytoskeletons [52]. The results of this study showed that actin cytoskeletal proteins were significantly associated with T cell infiltration, and TLN1 and CAPZB showed the most significant associations. Moreover, when immune cells attack cancer cells, the cancer cells can promote waveform protein expression and reorganize actins to evade immune cytotoxicity [53]; meanwhile, the actin cytoskeleton converts chemical signals from cell surface receptors into mechanical work, and mechanical stimuli indirectly affect antitumor immune responses via mesenchymal cells [54].

Filamin is a family of actin-binding proteins, including FLNA, FLNB, and FLNC, that have structural and scaffolding functions and are involved in various cellular processes, such as migration, cell adhesion, differentiation, proliferation, and transcription [55]. Furthermore, in this study, FLNA was significantly positively correlated with NKT cells in PRAD and DC cells in THCA. This suggests that high FLNA expression may activate and recruit NKT cells in PRAD in addition to promoting an increase in DCs and enhancing their function in THCA, which may increase the efficiency of antigen presentation and T-cell activation and thus enhance the antitumor immune response.

In this study, FLNA and FLNB showed a high mutation rate in UCEC and SKCM. FLNA is known to play a dual role in tumors. When located in the cytoplasm, full-length FLNA promotes tumor formation by interacting with signaling molecules that are closely linked to tumor invasion and metastasis. However, cleaved FLNA fragments located in the nucleus interact with transcription factors to reduce cancer invasion. Moreover, FLNA function is essential for anchoring receptors to the actin cytoskeleton and forming multiprotein complexes [56,57,58]. Therefore, it plays a crucial role in antitumor pathways. Previous studies have suggested that the development of drugs that target FLNA and cause their cleavage and subsequent localization to the nucleus may represent a novel and promising cancer treatment research area [58]. In prostate cancer therapy, long-term androgen deprivation leads to increased FLNA expression; however, once FLNA is localized to the nucleus, it can enhance the responsiveness of castration-resistant prostate cancer cells to androgen deprivation therapy by inducing apoptosis [59,60]. Cancer cells can effectively resist drug action by impairing drug–target interaction through acquired mutations at important drug-binding sites. Mouse NIH/3T3 cells expressing mutant actin proteins were more resistant to paclitaxel and vinblastine [61]. The high mutation rate of the actin-binding FLNA and FLNB proteins may also be related to drug sensitivity and resistance, but further research is needed to confirm this.

Genes involved in signaling pathways or cellular processes related to a drug’s mechanism of action can affect drug sensitivity. In this study, most actin cytoskeletal genes showed a significant negative correlation with the IC50 of XAV939. XAV939 is an inhibitor of tankyrases, which act as signaling and cytoskeletal proteins and can antagonize Wnt/β-catenin signaling [62]. XAV939 may participate in tumor cell signaling by regulating Wnt/β-catenin signaling [63], and it may also have anti-tumor effects by regulating cytoskeletal proteins.

Taken together, disulfidptosis-related actin cytoskeleton proteins are closely linked to tumor prognosis, the immune microenvironment, and drug resistance; regulating the actin cytoskeleton structure may be an effective cancer treatment strategy. Furthermore, combining immunotherapy with drugs based on actin cytoskeleton proteins may facilitate the therapeutic outcome of tumors. In addition, actin cytoskeleton proteins are mechanoresponsive proteins that serve as key components in cancer cell survival by adapting to different mechanical microenvironments [64,65,66]. Therefore, in the development of antitumor drugs targeting cytoskeletal proteins, compounds that modify the mechanoresponsiveness of cancer cells by altering their actin-binding activity may provide an additional therapeutic strategy [66].In future studies, we must first obtain direct evidence that actin cytoskeletal proteins can modulate tumor cell death by regulating disulfidptosis. In addition, further studies must demonstrate the effect of disulfidptosis-related genes on antitumor drug resistance and sensitivity. The development of targeted drugs based on these genes and their synergistic effects with immunotherapies must be explored in greater detail in the future.

In conclusion, this study presents a comprehensive overview of disulfidptosis- and actin cytoskeleton-related genes in pan-cancer. These genes are known to play a key role in the pathogenesis and progression of certain types of cancer, through their abnormal expression or mutation. They are also involved in other cancer-related pathways associated with tumor microenvironment and drug resistance. Given their potential value in cancer therapy, the disulfidptosis- and actin cytoskeleton-related genes could serve as prognostic and therapeutic biomarkers for various cancers. Thus, this study sheds light on their clinical significance and therapeutic potential for future research and treatment strategies.

## Supplementary Material

Supplementary Figure

Supplementary Table 1

Supplementary Table 2

Supplementary Table 3

Supplementary Table 4

Supplementary Table 5

Supplementary Table 6

Supplementary Table 7

Supplementary Table 8

Supplementary Table 9
