# Supplementary material for: Pan-cancer and single-cell analysis of actin cytoskeleton genes related to disulfidptosis
Source: Open Med (Wars). 2024 Mar 30;19(1):20240929. doi: 10.1515/med-2024-0929 (PMC10997004; doi:10.1515/med-2024-0929)
Supplement: Supplementary Figure [file med-2024-0929-sm.pdf]

Supplementary material

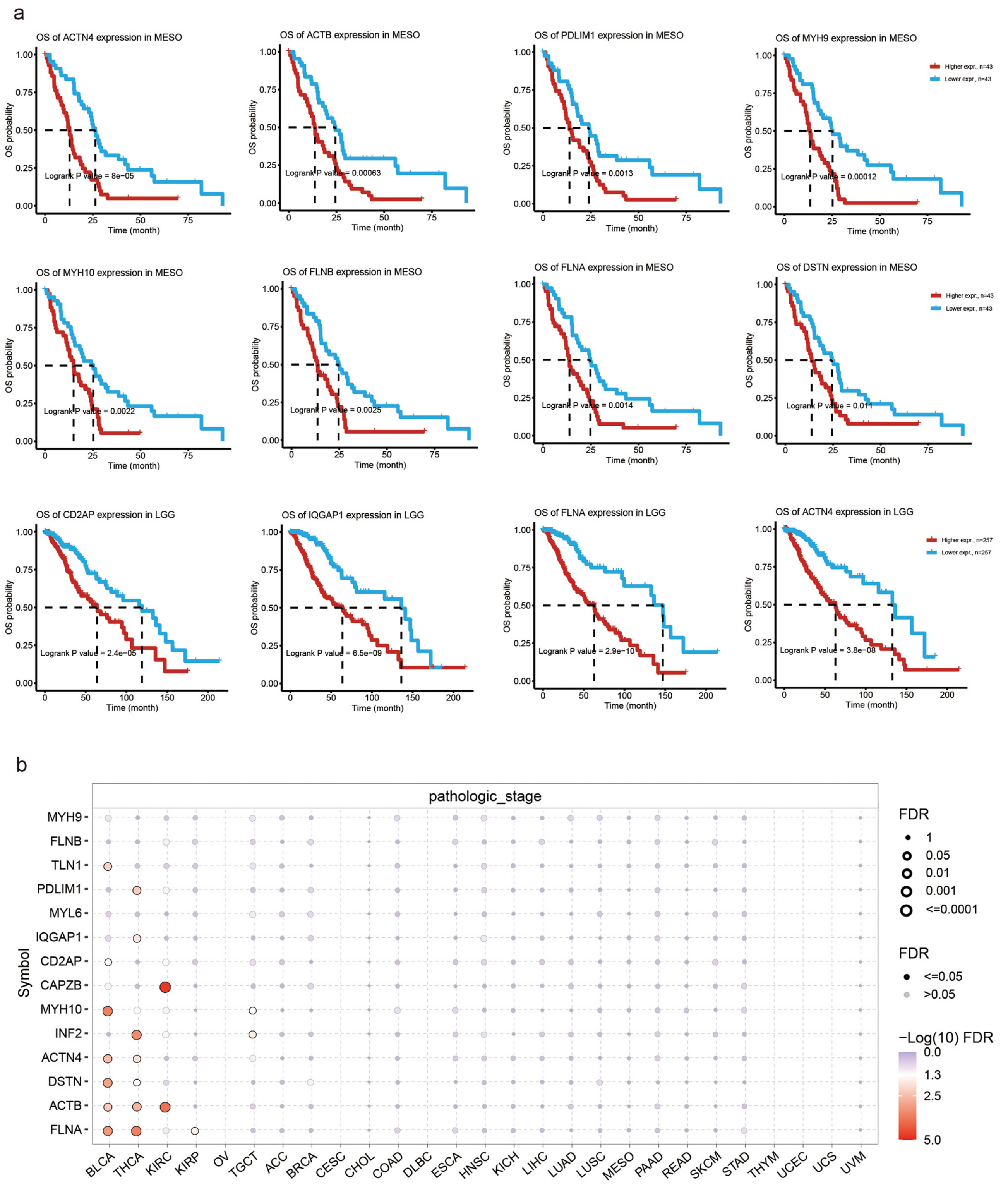

Figure S1: (a) Survival analysis based on a single gene expression level; (b) The correlation between disulfidptosis-related gene expression with cancer pathological stage.

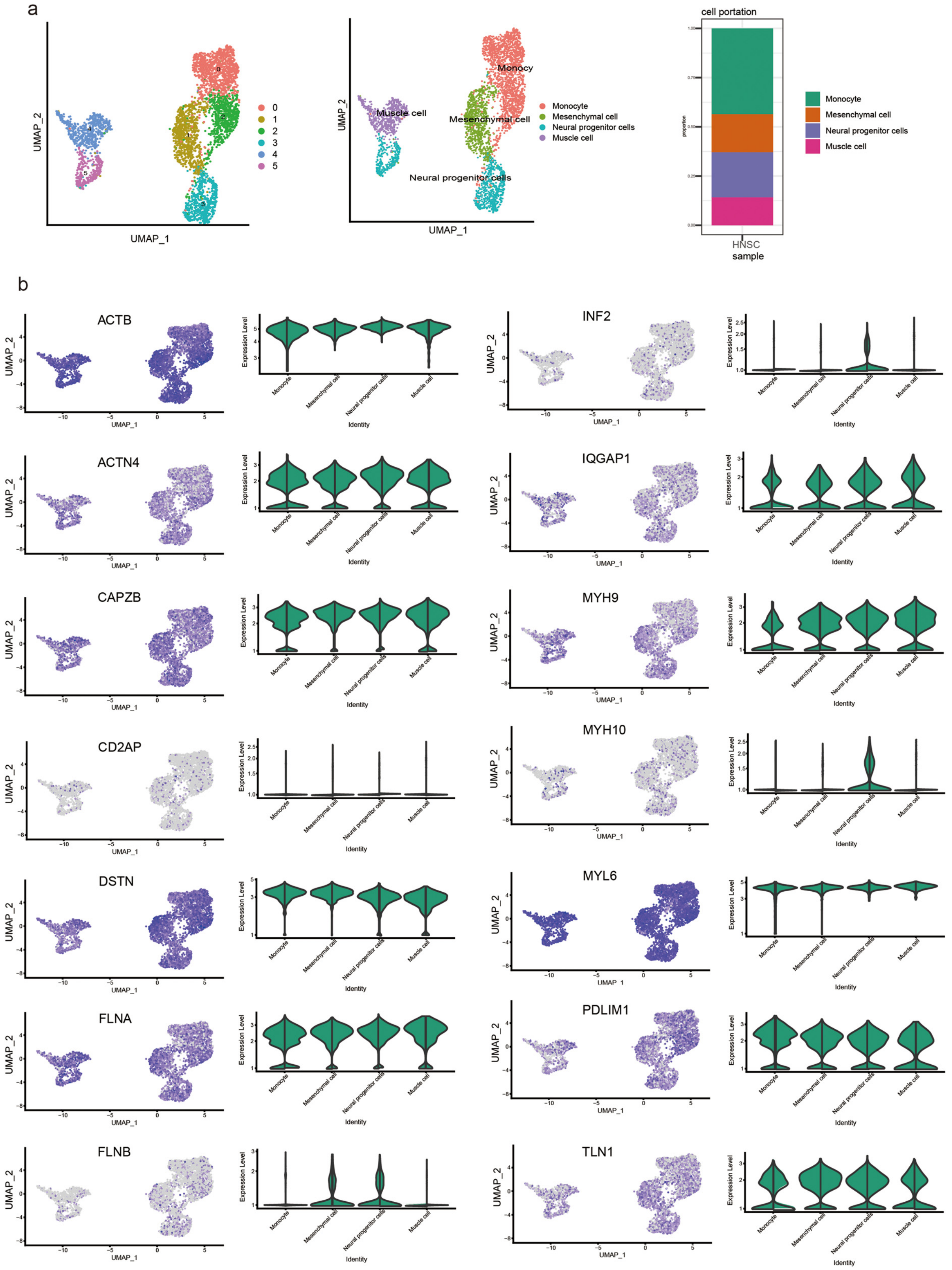

Figure S2: Single-cell sequencing analyzing the disulfidptosis-related gene expression in HNSC cells. (a) Cells were grouped and identified; (b) the expression level of disulfidptosis-related genes in different cell clusters.

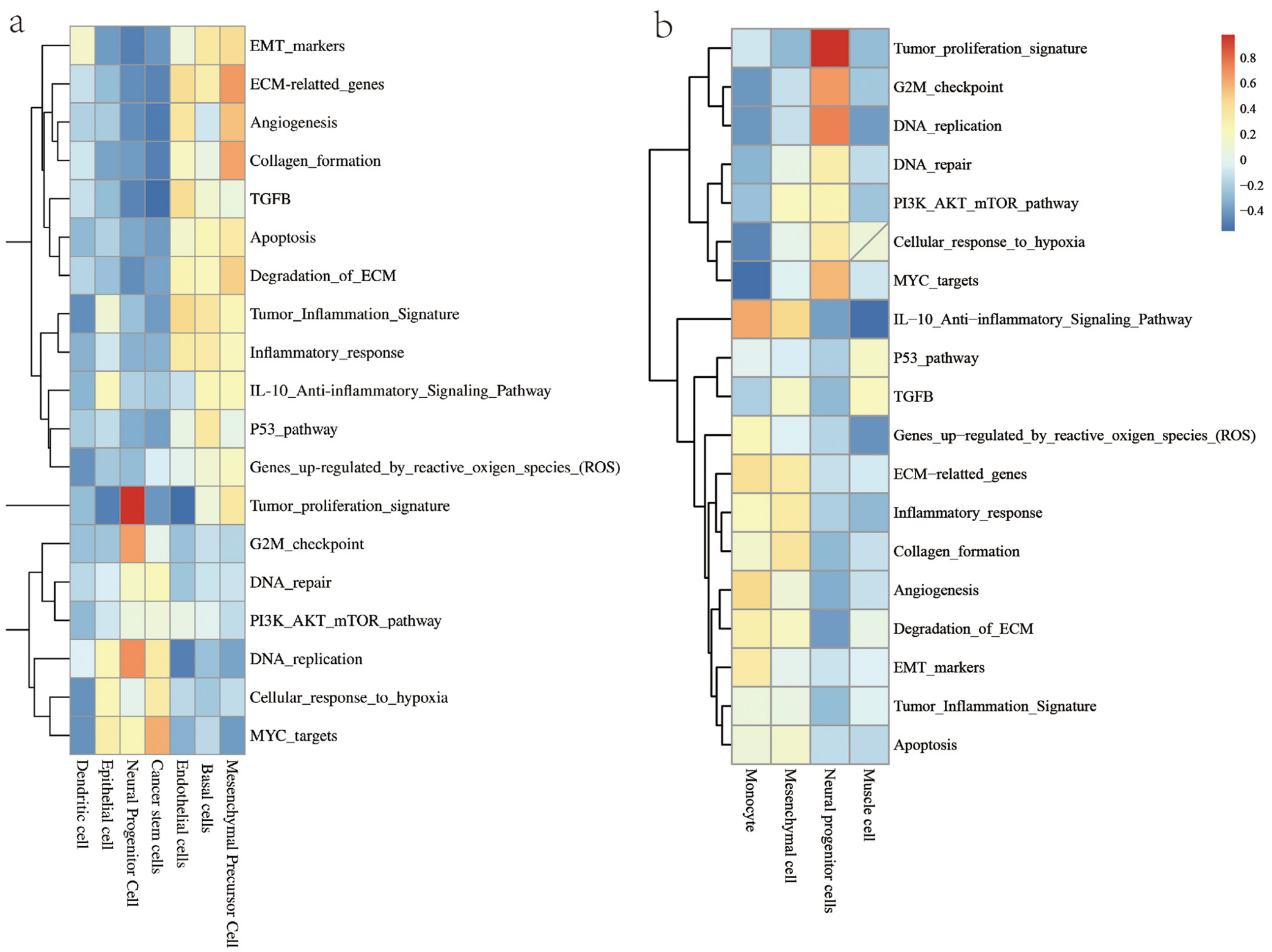

Figure S3: Heatmap of score in each pathway in different cell subsets of BLCA (a) and HNSC (b). Each column represents a diverse group or a distinct cell subpopulation, and each row represents a pathway, with more red colors representing higher scores and more blue colors representing lower scores.

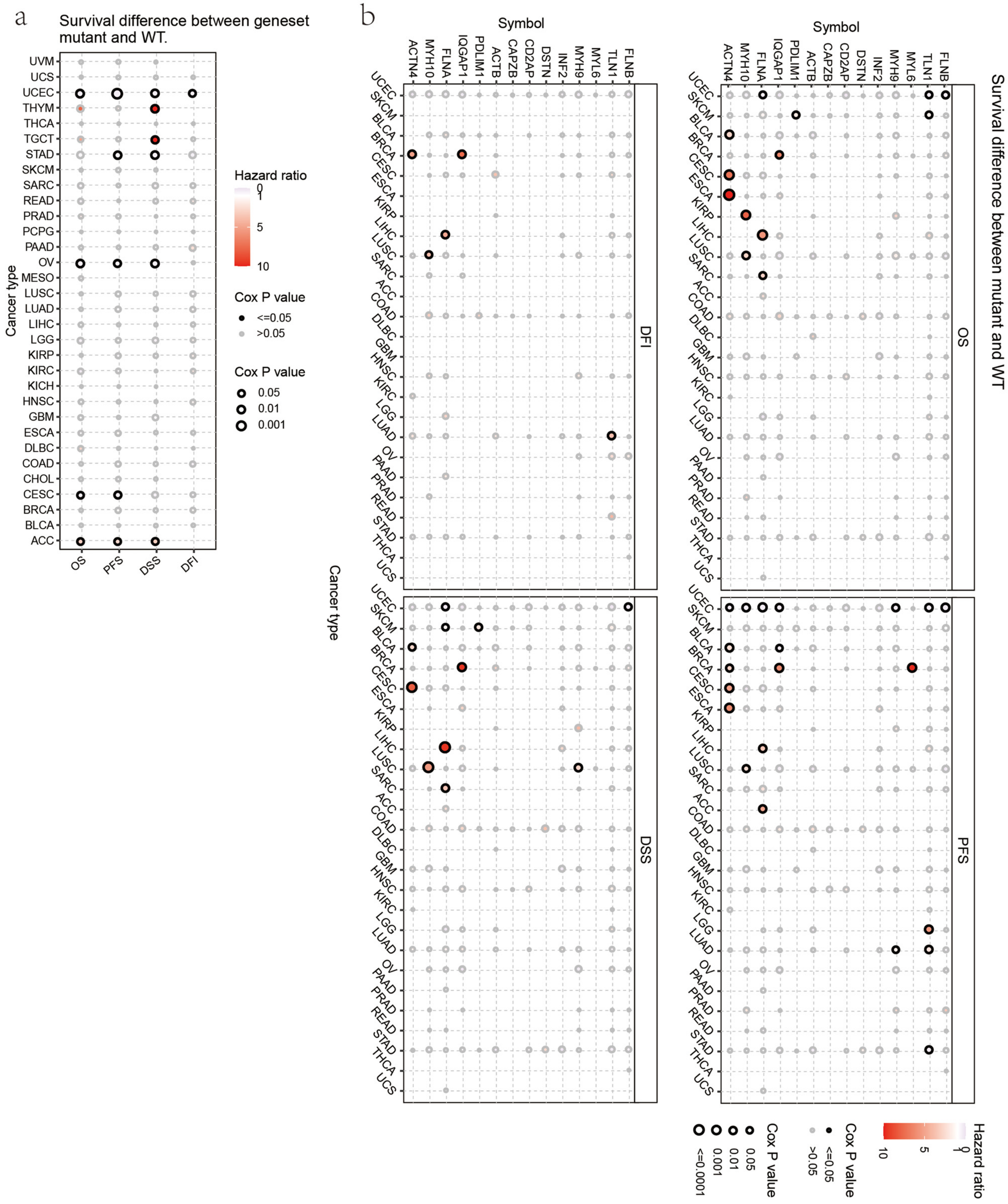

Figure S4: (a) The correlation between SNV with survival; (b) summarizes the survival difference between gene set mutant (deleterious) and wide type in the selected cancers.

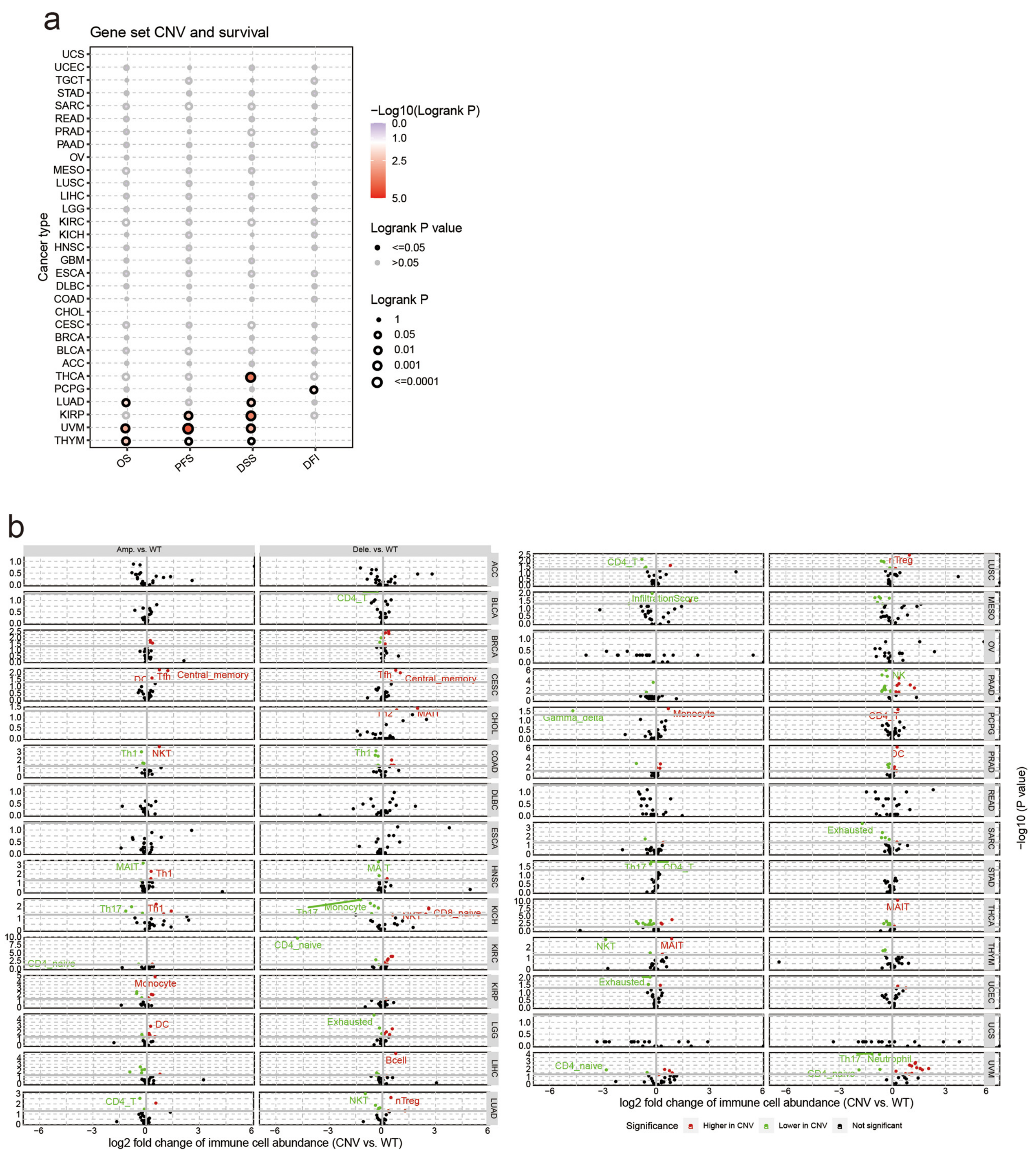

Figure S5: (a) summarizes the profile of survival between gene set CNV groups in the selected cancers; (b) summarizes the difference of immune infiltration between gene set CNV groups.
